# How Molar Mass, Acid Type, and Coagulation Bath Composition Influence Coagulation Kinetics, Mechanical Properties, and Swelling Behavior of Chitosan Filaments: A Full Factorial Approach

**DOI:** 10.3390/polym17070927

**Published:** 2025-03-29

**Authors:** Henrique Nunes da Silva, Milena Costa da Silva Barbosa, Matheus Ferreira de Souza, Athirson Mikael de Sousa Lima, Rafaella Resende de Almeida Duarte, Rômulo Feitosa Navarro, Suédina Maria de Lima Silva, Marcus Vinícius Lia Fook

**Affiliations:** 1 Postgraduate Program in Materials Science and Engineering, Department of Materials Engineering, Federal University of Campina Grande, Campina Grande 58429-900, PB, Brazil; milecost@hotmail.com (M.C.d.S.B.); matheusferza@gmail.com (M.F.d.S.); athirson.mikael.99@gmail.com (A.M.d.S.L.); rafaella.resende@certbio.ufcg.edu.br (R.R.d.A.D.); suedina.maria@professor.ufcg.edu.br (S.M.d.L.S.); 2 Materials Engineering Academic Unit, Federal Universisty of Campina Grande, Campina Grande 58249-900, PB, Brazil; romulonavarro13@gmail.com

**Keywords:** chitosan, wet spinning, coagulation process, mechanical properties, swelling behavior, full factorial design

## Abstract

In this study, a full multilevel factorial design (2^1^ × 3^1^ × 2^1^) × 2 was conducted to investigate the effects of molar mass of chitosan (CS), the type of acid used for dissolution, and the composition of the coagulation bath on the coagulation, mechanical properties, and swelling of the filaments. The results showed the statistical significance of the factors in the characteristics of these filaments. The coagulation followed Fick’s second law of diffusion, with an increase in the chitosan molar mass reducing the coagulation rate, as did the use of acetic acid instead of lactic acid. CS with higher molar mass produced filaments with larger diameters, but without a proportional increase in tensile strength. Swelling was influenced by the acid and composition of the coagulation bath. The interaction of CS with acid and the CS molar mass factor were the terms of greatest statistical significance. Crystallinity was higher for samples dissolved in aqueous solutions of acetic acid and coagulated with ethanol, while lactic acid induced greater structural disorder. Samples coagulated with ethanol presented more homogeneous surfaces, while methanol resulted in rougher filaments. These findings emphasize the critical role of processing conditions in tailoring the properties of CS filaments, providing valuable insights for their optimization for biomedical applications.

## 1. Introduction

Chitosan (CS), a semisynthetic polysaccharide derived from chitin, has become promising in the biomedical field due to its biocompatibility, biodegradability, and antimicrobial and healing properties [1,2,3,4]. These characteristics make CS widely used in pharmaceutical applications, tissue engineering, wound dressings, and controlled drug release systems [5,6,7,8,9,10]. One of the main advantages of this biopolymer is its solubility in aqueous acidic solutions. It can be processed into different physical forms, such as hydrogels or membranes [11], micro and nanoparticles [12,13], nanofibers [14], films [15], lyophilized scaffolds [16], woven meshes [17], and filaments [18,19]. However, its solubility can be influenced by several factors, such as deacetylation degree, solution pH, molar mass, solution temperature, and type of acid, among others [20].

CS filaments are usually produced by wet spinning, where the fibers are obtained by extrusion of viscous solutions in a coagulation bath. The precipitation or coagulation of the solution in the form of fiber involves an acid-base neutralization reaction and a process of diffusion and mass transport into the filament [20,21]. Among the coagulation media used, sodium hydroxide (NaOH) solutions stand out due to their efficiency in filament formation [18,19,22,23,24]. After the coagulation process, the filaments are washed and dried, where the washing bath can be carried out in distilled water [22] or aqueous solutions of methanol or ethanol [25].

The CS coagulation mechanism is complex and not fully understood, since it depends on several parameters, such as polymer concentration, degree of deacetylation, average molecular mass, type of solvent, nature and concentration of the coagulation agent, and the temperature of the coagulation bath [26]. Literature reports that the physical, mechanical, and biological properties (rate and mechanisms of reabsorption) of CS-based hydrogels are determined, in part, by the coagulation kinetics [27,28]. However, there are still significant gaps in the understanding of the coagulation step, which is essential for obtaining filaments with reproducible and controlled properties.

Studies such as those by Knaul and Creber [29] analyzed the coagulation of chitosan solutions in aqueous acetic acid, seeking to define optimal conditions for the formation of filaments with specific characteristics. Mohammadkhani et al. [30] investigated the influence of different coagulating agents, such as sodium hydroxide (NaOH) and mixtures with ethanol, demonstrating that the type of coagulant significantly impacts the mechanical and morphological properties of the filaments. Additionally, in Maevskaia et al. [31], work variables such as CS molar mass, polymer solution feed rate, and elongation rate were explored, showing that an increase in molar mass improves the structural integrity of the filaments. However, to the best of our knowledge, no studies have been reported that apply an experimental design to evaluate the influence of several independent factors simultaneously on the properties of CS filaments.

Design of Experiments (DOE) has become an essential tool for the optimization of complex manufacturing processes, allowing the identification and quantification of the effects of different variables on response variables. This methodology is widely used in different contexts to identify the ideal conditions that maximize the performance of processes or products. DOE makes it possible to reduce the number of experiments required, while concurrently providing detailed information on the impact of independent variables, based on a systematic, efficient, and statistically rigorous approach [32,33,34]. Its application may be relevant in multivariate processes, such as the coagulation of CS solutions for the formation of filaments and evaluation of their properties, where multiple parameters need to be controlled simultaneously to ensure desirable properties in the final product [35,36].

CS filaments are reported to have low to moderate mechanical properties, which limit their application [37,38,39,40]. Different approaches have been proposed to improve this aspect, using chemical modifications or physical treatments. Chemical modifications are generally simple, due to the large availability of reactive groups in the chitosan chains (amines and hydroxyls); however, these modifications can cause cytotoxic effects [37,41]. The routes used include crosslinking with epichlorohydrin [42], immersion in solutions containing phosphate and phthalate ions [22], modification with formic acid [39], fiber acetylation [43], re-annealing [44], reinforcement with chitin nanocrystals [40], and variation in solvent concentrations, coagulation bath composition, and drying under stretching [37]. In this sense, the application of a full factorial design to investigate the coagulation rate of spindle solutions and optimize the properties of CS filaments is a promising alternative to the approaches already reported in the literature.

This study aimed to apply an experimental design ((2^1^ × 3^1^ × 2^1^) × 2) to investigate how independent variables, such as the CS molar mass, the type of acid used in the dissolution, and the composition of the coagulant bath, influence the coagulation kinetics, mechanical properties, and swelling of the filaments obtained.

## 2. Materials and Methods

### 2.1. Materials

Chitosan (CS), medical degree, 96% deacetylated, and different molar mass (83, 155, and 200 KDa) was produced by CERTBIO (Laboratory of Biomaterials Assessment and Development from Northeast, Campina Grande, PB, Brazil). Lactic acid 85%, acetic acid 99.8%, sodium hydroxide, and methanol were bought from Neon (Suzano, SP, Brazil). Ethanol was acquired from Honeywell (Barueri, SP, Brazil). Phosphate saline buffer and lysozyme were obtained from Sigma Aldrich (Rio de Janeiro, RJ, Brazil).

### 2.2. Methodology

#### 2.2.1. Formulation of Spinnable Chitosan Solutions

CS solutions were prepared in accordance with factorial design conditions, varying molar mass, and acid solvent. To dissolve the polysaccharide, the volume of acid stoichiometrically related to the amine groups of CS was used (0.250 M). CS was dissolved in aqueous solutions of lactic or acetic acid, using an amount of polymer necessary for a final concentration of 4% (*m*/*v*). The mixtures remained under mechanical stirring for 2 h at 25 ± 1 °C and were centrifuged at 3600 rpm for 5 min to remove air bubbles. The coagulation baths were formed by 70% 0.5 M aqueous sodium hydroxide solution and 30% methanol or ethanol, according to the factorial design. Deionized water was used for the aqueous solutions.

#### 2.2.2. Obtaining Chitosan Filaments

The methodology for preparing CS filaments was adapted from those previously reported [17,18,45,46]. A syringe containing 4% *w*/*v* CS solution was fixed in an infusion pump to promote the extrusion of the polymeric solution in the coagulation bath, under constant flow (45 mL/h) and 1 mm diameter outlet tip. The parameters of the wet-spinning process, such as CS molecular weight, acid solvent, and composition of the coagulation bath, were followed according to the combinations of factor levels in the experimental design. The CS filaments remained in the coagulant solution for 10 min and, finally, were taken out, washed with distilled water until a pH near 7 was achieved, and subsequently dried at 60 ± 2 °C for 1.5 h in an oven.

#### 2.2.3. Full Factorial Experiment Design

Among the various factors that can affect the coagulation rate and properties of chitosan fibers, the molecular weight, acid type, and composition of the coagulation bath were selected. Thus, a multilevel full factorial design (FFD) was used to conduct the experiments. Table 1 shows the independent parameters and their levels.

A total of (2^1^ × 3^1^ × 2^1^) × 2 = 24 combinations/treatments were evaluated, corresponding to 12 systems in duplicate. The experiment was run in random order and without blocking, as shown in Table 2.

Experimental design and statistical treatment of the data were carried out in Minitab 19 software. The response variables evaluated were coagulation rate (mm/s^1/2^), tensile strength (MPa), Young’s modulus (GPa), and swelling degree in 24 h (%). Numerical results are presented as means and their respective standard deviations. For *p*-value < 0.05, the difference between the means was considered significant.

#### 2.2.4. Coagulation Rate Measurement

**Figure 1:**
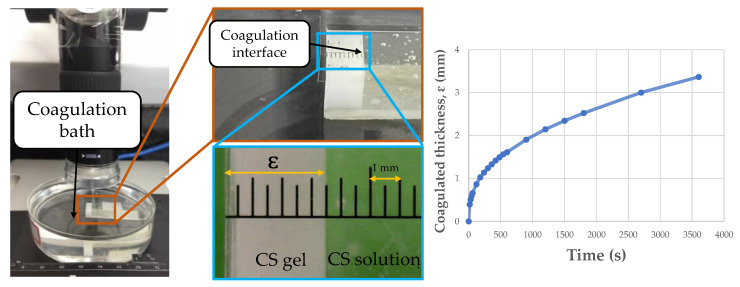
Coagulation system used to evaluate coagulation kinetics.

The coagulation experiments were performed with CS solutions 4% *w*/*v*, dissolved in acetic or lactic acid, and coagulated in NaOH/ethanol or NaOH/methanol. The experiments were conducted based on an adaptation of the methodology described by Enache, David, Puaux, Banu, and Bozga [28]. A transparent quartz cell with a height of 4.5 cm, a wall thickness of 1 mm, and inner width of 1 cm was filled with the CS solution (3 mL) and fixed with double-sided tape on the bottom of a Petri dish (diameter of 9 cm and height of 2.5 cm), where a 100 mL coagulation bath was filled. The coagulation rate was investigated by measuring the time evolution of the coagulated thickness inside the quartz cell. To do this, images of the coagulated layer with different times were captured, using the Digital Viewer software (version 3.1.07) and a portable USB digital microscope (Czpan, Beijing, China) with a 0.3 m CMOS image sensor, with focus ranges between 15 and 40 mm. The experimental arrangement is shown in Figure 1.

In coagulation experiments, the temporal advance of the boundary between the coagulated and soluble phases was recorded for 1 h, at 25 ± 0.5 °C and 54 ± 2% relative humidity. Thus, the coagulated thickness profile (ɛ, mm) with time (s) was obtained for each of the 12 systems under study.

Diffusion of the small molecules and ions on CS-based hydrogels is adequately described by the Fickian diffusion model [27,28,29,47,48,49]. The studies by Knaul and Creber [29], Araiza et al. [47], and Enache et al. [28] demonstrated that the coagulation process of CS solutions occurs through the diffusion of OH- ions in the polymer matrix. Furthermore, since this process is governed by Fick’s second law, it implies that the thickness of the coagulated CS solution (mm) must be proportional to the square root of time (t^1/2^). It follows that ɛ/t^1/2^ is a constant parameter, a term defined by Paul [50] as the “coagulation rate”. In this sense, the coagulation rate was obtained by the slope of the linear regressions of the coagulated thickness (mm) in function of the square roof of the time (t^1/2^).

#### 2.2.5. Swelling Degree

The swelling degree of CS filaments (samples with a length of 50 mm, *n* = 2 to each of the FFD treatment) was evaluated in 5 mL phosphate buffer saline solution (PBS, pH 7.4). The samples were dried at 50 °C for 6 h, weighed (W_0_), and then conditioned at 37 ± 0.5 °C in PBS. After 24 h, the samples were taken from the swelling medium, gently dried with absorbent paper, and weighed (W_24h_). The swelling degree at 24 h was determined according to Equation (1):

(1) $${\mathrm{SD}}_{24h}\left(\%\right)=\frac{W_{24h}-W_{0}}{W_{0}}\;\times \;100$$

where W_24h_ and W_0_ represent the mass of swollen and dried state samples, respectively.

#### 2.2.6. Instrumentation

Mechanical properties of the CS filaments were evaluated by uniaxial tensile testing. Samples (*n* = 10) were analyzed using a universal testing machine (Instron, Model 6633-Norwood, MA, USA), with a 500 kN load cell, displacement speed of 120 mm/min, and 100 mm claw distance. X-ray diffraction (XRD) patterns were used to determine the crystallinity index (CI) as described by da Silva et al. [18]. A diffractometer (Shimadzu model XRD-7000—Shimadzu, Tokyo/Kyoto, Japan) with Ni-filtered Cu-Kα radiation, a scattering range of 5° < 2θ < 40°, resolution of 0.02°, scanning speed of 1°/min, 40 kV of voltage, and 30 mA of current was used. The CS filaments were arranged in parallel and then subjected to the assay. Fourier transform infrared spectroscopy (FTIR) spectra of the samples were obtained in the range of 4000–650 cm^−1^, resolution of 4 cm^−1^ and 32 scans on a Perkin Elmer spectrometer (Spectrum 400 FT Mid-IR, Waltham, MA, USA) coupled with an attenuated total reflectance (ATR) accessory. Morphology and diameter of the CS filaments were evaluated by scanning electron microscopy (SEM), using a Hitachi model TM-1000 (Chiyoda, Tokyo, Japan) electron microscope with a maximum magnification of 10,000×, depth of focus of 1 mm, resolution of 30 nm, 15 KV, low vacuum and varied pressure (1 to 270 Pa), and a thin layer of gold as a metallic coating.

## 3. Results and Discussion

### 3.1. Coagulation Measurements of CS Solutions

Coagulation of the CS solution occurs due neutralization of the protonated amine groups (-NH_3_^+^) by the OH^−^ ions from the sodium hydroxide on the coagulation bath. This process is governed by diffusion of the OH^−^ ions through the coagulated hydrogel layer. Factors such as specimen mobility, solution viscosity, and molecular interaction affect this diffusion process [28,29,51]. Figure S1 shows the coagulation profile of the 12 different systems, with variations in the CS molecular wight, acid solvent, and composition of the coagulation bath. Coagulation profile is present as the coagulation thickness, ɛ, (mm) in function of the time (s). With the diffusion of the OH^−^ ions into the coagulation cell, ɛ grows and becomes an additional barrier for the diffusion of ions. In this sense, the rate that the ɛ grows decreases with time, resulting in a logarithmic behavior [27,28]. This behavior could be noticed for all studied systems; however, in some cases, the effect of molecular weight appears to be negligible compared to other factors.

**Figure 2:**
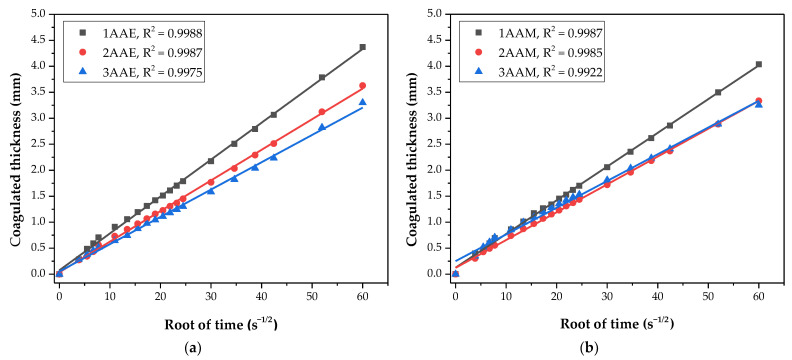

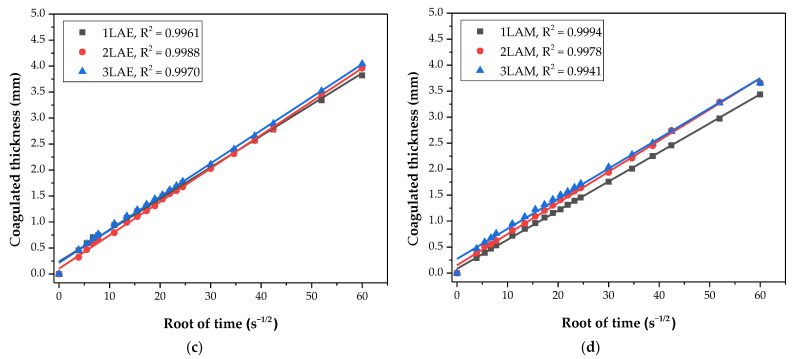
Adherence to Fick’s law of coagulated thickness evolution compared to the square root of time for the evaluated systems. Samples were prepared with chitosan of variable molar mass (3, 2, and 1) and (**a**) acetic acid and ethanol, (**b**) acetic acid and methanol, (**c**) lactic acid and ethanol, and (**d**) lactic acid and methanol.

In Figure 2, the adequation of the coagulation process of the 12 different systems corresponds with Fick’s second law, when comparing ɛ (mm) with the square root of time (s^1/2^). For all of the systems, the determination coefficient (R^2^) of the data linear regression is greater than 0.99, which shows an excellent fitting of the CS coagulation process to the Fickian diffusion model [27,29].

The coagulation rate was defined as the angular coefficient (slope) of the data linear regression [50] (Supplementary Material, Table S2). The mean coagulation rate of the systems is shown in Table 3.

The samples dissolved in acetic acid and coagulated in NaOH/ethanol solutions showed that the coagulation rate has an inverse relationship with the molecular weight of the CS, with separation and different slopes between the regressions. For these samples, the coagulation rate increases with decreasing molecular weight, a result that is reported by Masaro and Zhu [51]. This is probably due to the viscosities of the solutions, which are fundamentally associated with the molar mass of the polymer [52,53]. The greater the molecular entanglement, and consequently the viscosity, the greater the barrier to the diffusion process of OH^−^ ions, resulting in a lower coagulation rate, as observed by Venault et al. [27] and Enache et al. [28].

**Figure 3:**
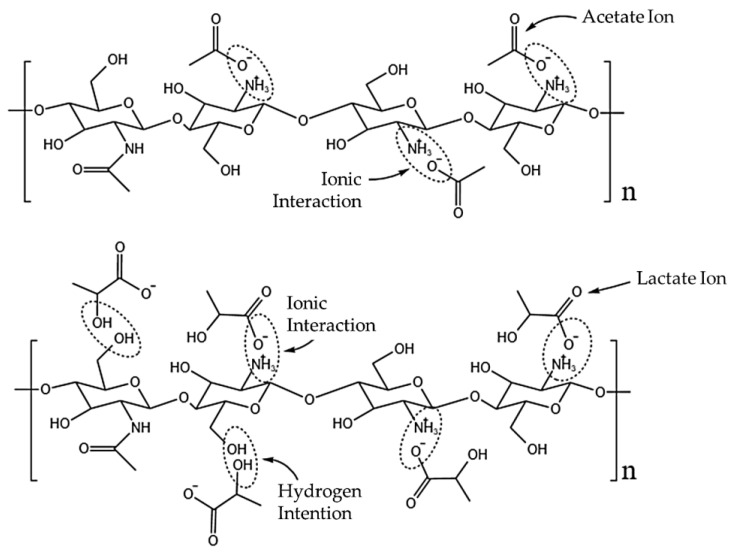
Possible interactions between the acetate and lactate anions with protonated chitosan.

The samples with acetic acid as a solvent and coagulated in NaOH/methanol (1AAM, 2AAM, and 3AAM) also presented a certain separation of curves. However, the samples prepared with lactic acid presented practically overlapping coagulation profiles, which can be explained based on the interaction between the protonated chitosan and the lactate anion, as illustrated in Figure 3.

The intermolecular interactions between chitosan and carboxylic acids are directly related to the length and complexity of the acid chain and, consequently, to the anion generated during its dissociation (Shamov et al.) [54].

Hamdine et al. [55] proposed that anions with smaller molecular volumes (such as the acetate ion) can form hydrogen bonds with chitosan more easily, due to the better approximation of potential hydrogen bonding sites. The observed effect is the stabilization of the physical network, resulting in more cohesive and higher-viscosity gels. Furthermore, the authors observed that lactic acid does not promote the gelation of chitosan, unlike acetic acid. This is probably the factor responsible for the increase in the coagulation rate when the acid used is changed from acetic to lactic. Protonated chitosan and acetate ions interact mainly in an ionic manner, through the NH_3_^+^ groups of chitosan and O^−^ of the anion (Figure 3). The interaction with the lactate ion can occur ionically or by hydrogen bonds since lactate retains a hydroxyl (OH) in its structure. Since the lactate molecule is not large enough to enable physical crosslinking between the chitosan chains, what occurs in practice is a plasticizing effect, with a reduction in interactions between the chitosan chains, since their interaction points (OH) are unavailable due to the interaction with lactate [53,56,57,58].

Soares et al. [57] reported that chitosan chains interact more strongly with hydroxylated acid anions. The authors observed that the lactate ion binds more strongly to the chitosan chains, promoting a more expressive reduction in pH and viscosity of the solutions, compared to chitosan solutions in acetic acid. This may justify the overlap in the coagulation rates of the systems with lactic acid.

### 3.2. Mechanical Properties of CS Filaments

The mechanical properties and diameters of the filaments obtained from the 12 systems under study are presented in Table 4. The filaments were analyzed for tensile strength, Young’s modulus, and strain at fracture.

The results of the tensile test demonstrate that the CS molar mass, the choice of acid as solvent, and the composition of the coagulant bath significantly influence the mechanical properties of the filaments obtained. In general, the increase in the CS molecular weight increased the diameter of the filaments. This effect may be related to the higher viscosity of the high-molecular-weight chitosan solutions, which favors the formation of thicker fibers during extrusion, due to the increased incidence of amine groups and the greater degree of entanglement of the polymer chains [59,60]. On the other hand, this increase in diameter did not result in a proportional gain in tensile strength. Filaments with larger diameters may present a less homogeneous distribution of molecular orientation and a lower degree of crystallinity, reducing mechanical strength [61]. In addition, the presence of internal porosity, which tends to be more pronounced in filaments with larger diameters, compromises structural integrity and can act as stress concentration points [62].

The choice of the type of acid used in the dissolution of chitosan had a significant impact on the mechanical properties of the filaments. Those prepared with lactic acid (LA) presented higher tensile strength and deformation capacity (%) compared to filaments prepared with acetic acid (AA). This effect was particularly evident in sample 1LAE, which presented the highest tensile strength of the study (154 MPa) and the highest elongation before rupture (5.7%). The mechanical superiority of filaments with lactic acid can be attributed to the more intense interaction between lactic acid and the chitosan structure, resulting in a more resistant structure [57].

The comparison between ethanol (E) and methanol (M) present in the coagulation bath directly impacted the tensile strength and stiffness of the filaments. The filaments prepared with ethanol generally presented higher tensile strength for the lower molecular weights, but this property was significantly reduced by increasing molecular weight. In samples with higher molar mass, the presence of ethanol can induce a less efficient reorganization of the polymer chains, resulting in less dense structures and, consequently, lower tensile strength. The filaments coagulated in the presence of methanol presented less variation in tensile strength with the increase in molecular weight. According to Sun et al. [63], the presence of alcohols can modify the structural organization of the polymer chains and the interaction between the charged groups, which can explain these mechanical variations.

### 3.3. Swelling Degree of CS Filaments

**Figure 4:**
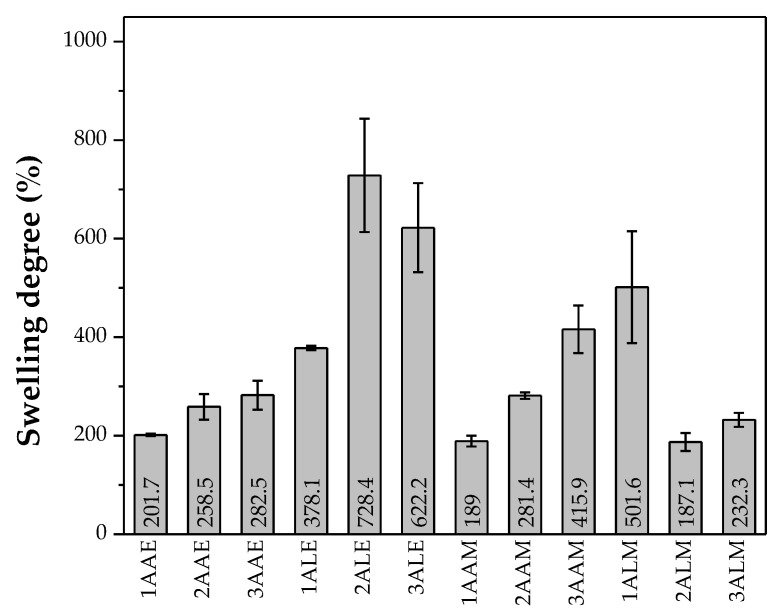
Average swelling degree exhibited by the evaluated systems.

Figure 4 shows the average results of the degree of swelling in 24 h obtained for the CS filaments in contact with phosphate buffer saline solution (PBS). All filaments showed high swelling capacity, absorbing more than their weight (individual values > 100%). CS has a highly hydrophilic structure. According to Mucha et al. [64], CS forms hydrogen bonds with water through its hydroxyl and amide groups, favoring greater water retention.

It is observed that the filaments prepared with lactic acid generally presented greater swelling compared to the filaments prepared with acetic acid. This behavior may be related to the more polar structure of the lactate counterion with the CS structure, promoting greater water absorption and expansion of the polymer matrix [57]. In addition, the interactions between CS and lactic acid may hinder the movements of the polymer chain segments, restricting the crystallization process and resulting in a more amorphous structure, which favors water absorption [65].

Furthermore, the filaments coagulated in ethanol showed greater swelling compared to those coagulated in methanol. This phenomenon can be attributed to the action of ethanol in the coagulation bath, which promotes increased water permeability, as reported by Liu et al. [66]. The filaments obtained with CS of higher molar mass showed greater swelling, although some exceptions were observed. This occurs because polymers of higher molecular weight have longer and more entangled chains, forming a structural network that retains water molecules more efficiently [67].

### 3.4. Analysis of the Factorial Design

#### 3.4.1. Multiple Regression

The linear factors (A, B, and C) and second- (A × B, A × C, and B × C) and third-order interactions (A × B × C) were adjusted using Minitab software, with the one-way ANOVA tool, for the response variables: coagulation rate, tensile strength, Young’s modulus, and swelling degree. Mathematical models are provided in the Supplementary Material (Table S3).

Table 5 presents the ANOVA results for the coagulation rate and tensile strength, while Table 6 presents the results for Young’s modulus and degree of swelling. To meet the adopted significance level of 95%, the *p*-value must be lower than 0.05 [32,68]. For all response variables evaluated, the models must present a *p*-value < 0.033, which means that at least one of the factors or interactions in the model has a significant effect on the response variables.

For the coagulation rate, 88.77% of the total variation is explained by the model. Three terms presented statistical significance (*p*-value < 0.002). The interaction A × B (CS molar mass × acid) contributed the most to the model, 48.10%, being the only interaction to present statistical significance. This indicates a highly significant interaction between factors A and B (CS molar mass and acid used, respectively), showing that the effect of A on the response depends on B and vice versa. In second place, there is factor A, with a 21.66% contribution, with the CS molar mass being one of the determining factors for the coagulation rate. The third significant factor with the greatest contribution was C (composition of the coagulating bath), with 14.95%. Of the individual factors, only B did not present statistical significance. The error represents 11.23% of the total variation.

The model explains 73.74% of the variation in tensile strength, with 32.57% of this variation attributed to the interaction A × C (molar mass of CS × composition of the coagulation bath). This interaction should be considered as a key element in the analysis since the effect of A on tensile strength depends on C and vice versa. The other terms of the model did not show statistical significance, with a *p*-value > 0.061. The error for this model was 26.26%.

For Young’s modulus, 81.11% of its variation can be explained by the model. The terms with the greatest contribution and statistical significance were the interactions A × C (CS molar mass × coagulation bath composition) with 38.85% and A × B (CS molar mass × acid) with 21.80%. Main factors (A, B, and C) and interactions with minor contributions (B × C, A × B × C) do not show statistical significance in isolation (*p*-value > 0.096), while the error represents 18.89% of the total variation.

The model for the degree of swelling describes 94.76% of its variation. Only factor A (molar mass of CS) was not significant, *p*-value = 0.078. Factor B and the interactions B × C and A × B × C presented the largest contributions to the model, 23.45, 20.37, and 20.87%, respectively. The error represents only 5.24% of the total variation, showing that most of the variability is captured by the model.

Table 7 presents the important parameters for evaluating the quality of the models adjusted to the response variables: Standard Error of the Estimate (S), Coefficient of Determination (R-sq), Adjusted R-squared (R-sq(adj)), Predicted Residual Error Sum of Squares (PRESS), Corrected Akaike Information Criterion (AICc), and Bayesian Information Criterion (BIC).

The model for swelling degree presents the highest R-sq (94.76%) and R-sq(adj) (89.95%), indicating the best fit to the data. The coagulation rate is the second-best model, with an excellent R-sq (88.77%) and the lowest S value. It presents excellent parsimony with the lowest AICc and BIC values. The Young’s modulus model is intermediate, with good R-sq (81.11%) but a significant drop in R-sq(adj) (63.80%). This may indicate that the model can be refined. Tensile strength is the weakest model, with the lowest R-sq (73.74%) and the largest difference about R-sq(adj) (49.68%), suggesting overfitting and low robustness.

The models were developed to determine the ideal molar mass, acid, and coagulation bath composition to minimize the coagulation rate and swelling degree and maximize the tensile strength and Young’s modulus.

#### 3.4.2. Pareto Chart of the Responses

**Figure 5:**
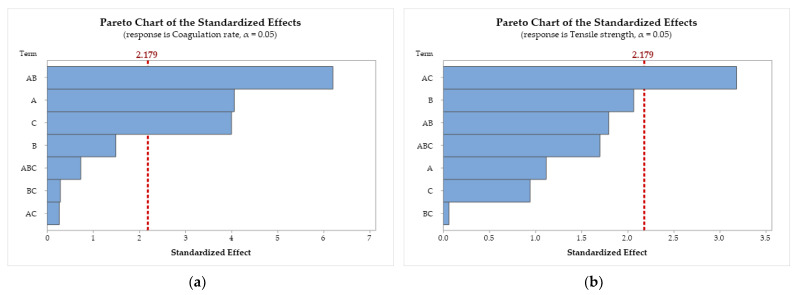

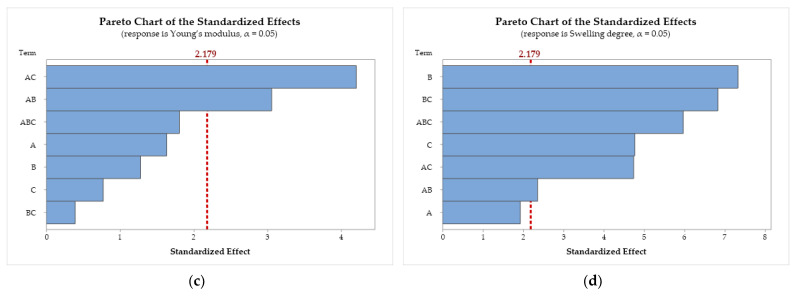
Pareto chart of the standardized effects for (**a**) coagulation rate; (**b**) tensile strength; (**c**) Young’s modulus; and (**d**) swelling degree. Chitosan molar mass (A), acid (B), and coagulation bath (C).

Pareto diagrams for the response variables are presented in Figure 5. The terms (factors and interactions) of the model are ranked in a graphical representation according to their significance, from most influential to least influential. The red dashed line (*p*-value = 0.05, for a 95% statistical significance level) represents the minimum magnitude of statistically significant effects [68,69].

Figure 5a demonstrates that the A × B interaction (CS molar mass × acid) has the largest standardized effect on the coagulation rate, followed by factors A (CS molar mass) and C (bath composition). Factor B and the interactions A × B × C, B × C, and A × C are statistically insignificant, as they do not cross the threshold.

In Figure 5b, only the interaction A × C (CS molar mass × bath composition) showed statistical significance in the standardized effects on tensile strength. For Young’s modulus (Figure 5c), the results indicate that the interactions A × C and A × B show the largest standardized effects. In Figure 5d, the results demonstrate that the individual factors B and C and the interactions A × B, A × C, B × C, and A × B × C show significant standardized effects on the swelling degree, with A being the only factor without statistical significance.

#### 3.4.3. Contour and Surface Plots of the Responses

**Figure 6:**
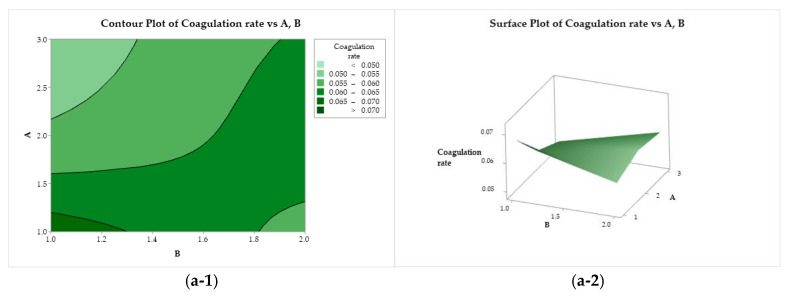

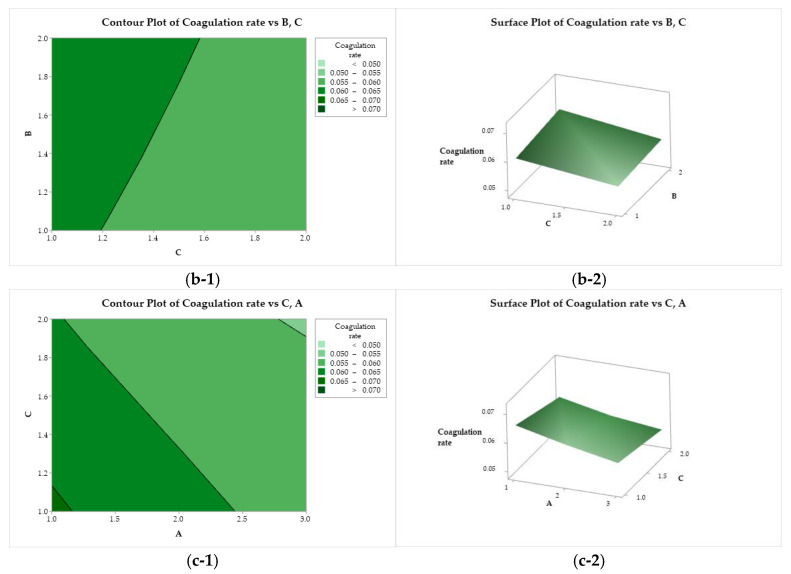
Contour (1) and surface (2) plots of the coagulation rate with variances of chitosan molar mass (A), acid (B), and coagulation bath (C).

The effects of the variation in the independent factors (A, B, and C) on the response variables can be evaluated using the contour plots and response surface tools. Figure 6(a-1, a-2) illustrate the behavior of the coagulation rate as a function of the CS molar mass (factor A) and acid used (factor B). The coagulation rate increases with the darkening of the color. The highest coagulation rate was observed for the combination of CS with a molar mass of 83 kDa (A = 1) solubilized with acetic acid (B = 1). This response variable is negatively affected when the CS molar mass increases, reaching its minimum when a high molar mass is used (200 kDa, A = 3). In Figure 6(b-1, b-2), the coagulation rate exhibits a practically flat behavior as a function of factors B (acid) and C (bath composition), where the use of ethanol results in higher coagulation rates. A similar behavior was observed when the response was evaluated as a function of C and A. The coagulation rate is maximized when using low molar mass chitosan and ethanol in the coagulant bath (A = 1, and C = 1), while the minimum is reached when using high molar mass CS and methanol (A = 3, and C = 2).

**Figure 7:**
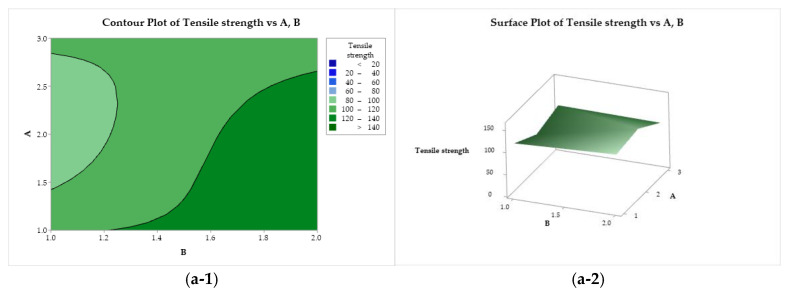

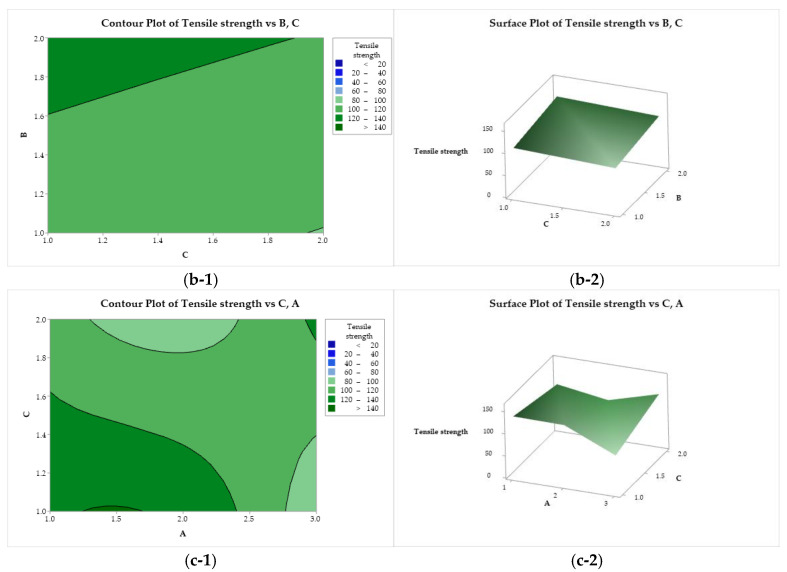
Contour (1) and surface (2) plots of the tensile strength with variances of chitosan molar mass (A), acid (B), and coagulation bath (C).

Figure 7(a-1, a-2) represent the tensile strength behavior as a function of factors A and B. Maximum strength is provided by the use of CS with low or medium molar mass (A = 1 or 2) solubilized with lactic acid (B = 1). On the other hand, the yarns obtained with CS of medium molar mass (A = 2) and acetic acid are not very strong. In Figure 7(b-1, b-2), it can be seen that the use of lactic acid (B = 2) and ethanol (C = 1) increases the strength of the filaments, while the use of CS of low molar mass and methanol tries to minimize this property. In Figure 7(c-1, c-2), it is possible to observe that the tensile strength exhibits a minimax behavior with a stationary point (saddle point) close to the upper right quadrant, and two maximum points ((A = 1, C = 1) and (A = 3, C = 2)) and minimum points ((A = 2, C = 2) and (A = 3, C = 1)).

**Figure 8:**
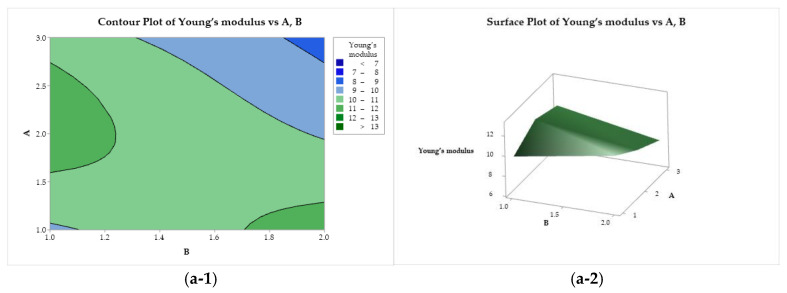

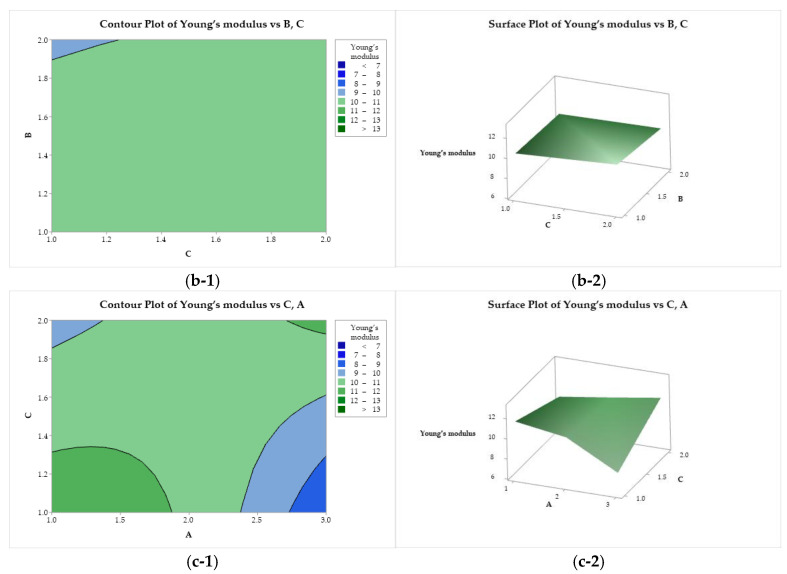
Contour (1) and surface (2) plots of the Young’s modulus with variances of chitosan molar mass (A), acid (B), and coagulation bath (C).

In Figure 8(a-1, a-2), Young’s modulus is presented as a function of factors A and B. A saddle point is observed near the center of the design, minimum points at (A = 3, B = 2) and (A = 1 and B = 1) and maximum points at (A = 1, B = 2), and at the center of the left edge (A = 2, B = 2). Young’s modulus presented a flat behavior as a function of B and C (Figure 8(b-1, b-2)), with a minimum for the use of lactic acid (B = 2) and ethanol (C = 1). A minimax pattern is observed for Young’s modulus as a function of A and C (Figure 8(c-1, c-2)), with a saddle point near the center of the graph. Two minima are observed, at (A = 3, C = 1) and (A = 1 and C = 2). The two maximum points are at (A = 1, C = 1) and (A = 3, and C = 2).

**Figure 9:**
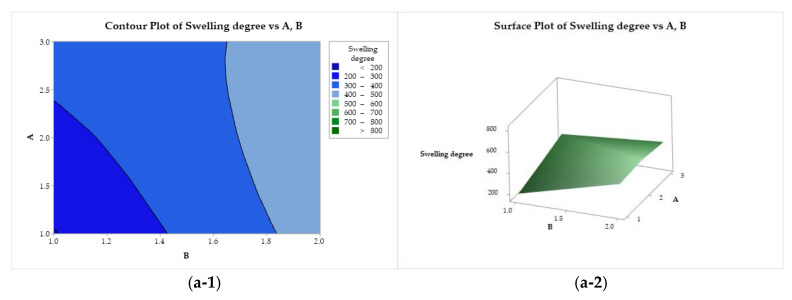

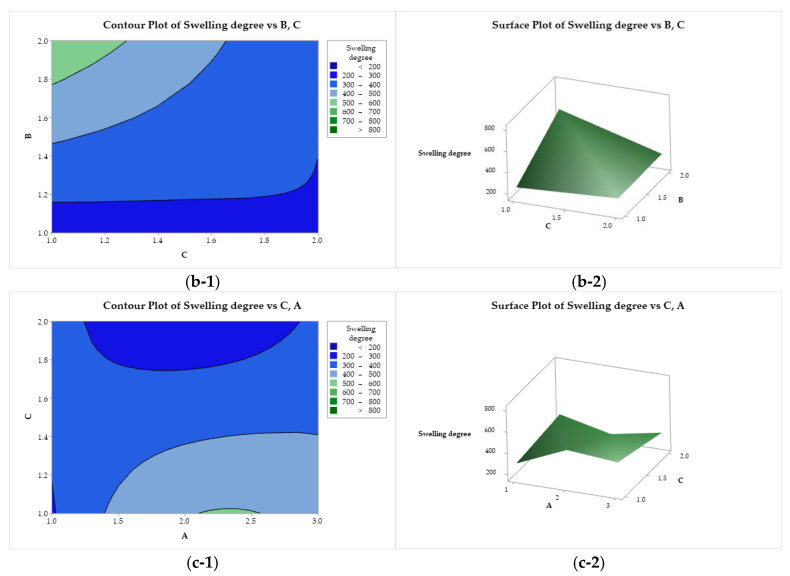
Contour (1) and surface (2) plots of the swelling degree with variances of chitosan molar mass (A), acid (B), and coagulation bath (C).

Figure 9 presents the contour and response surface plots for the degree of swelling as a function of factors A, B, and C. Figure 9(a-1, a-2) show that the degree of swelling is maximized for all CS molar mass levels (factor A) when lactic acid (B = 2) is used. Minimization is achieved when factors A and B are adjusted to lower levels (low molar mass and acetic acid, respectively). As a function of B and C (Figure 9(b-1, b-2)), the degree of swelling exhibits an ascending crest behavior, with minimization with the use of acetic acid (B = 1), regardless of the composition of the coagulation bath, and maximum for the use of lactic acid (B = 2) and ethanol (C = 1). A saddle point is observed for the degree of swelling as a function of A and C (Figure 9(c-1, c-2)). This property is minimized with the use of medium molar mass CS (A = 2) and methanol in the coagulation bath (B = 2), whereas it is maximized for medium and high molar mass CS (A = 2 and 3) combined with ethanol (C = 1).

#### 3.4.4. Main Effects Plot of the Responses

**Figure 10:**
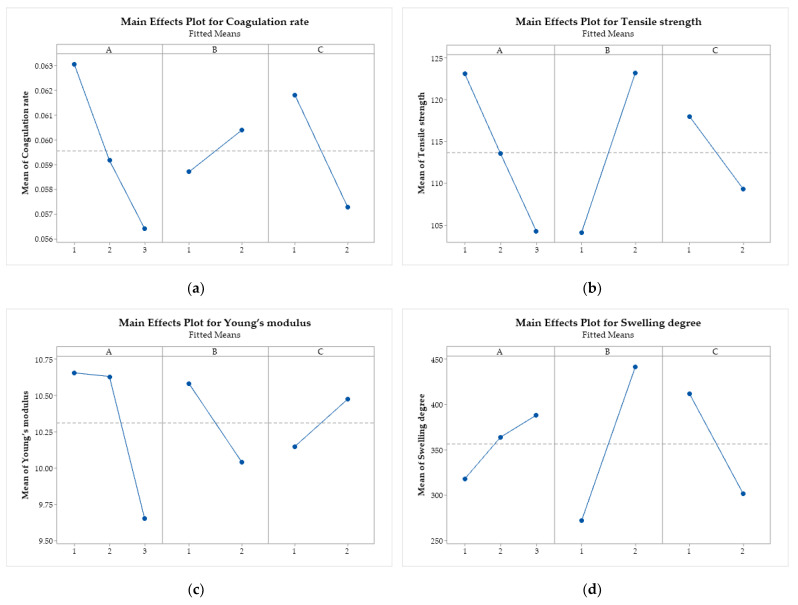
Main effects plot for (**a**) coagulation rate; (**b**) tensile strength; (**c**) Young’s modulus; and (**d**) swelling degree. Chitosan molar mass (A), acid (B), and coagulation bath (C).

Figure 10 presents the graphs of the main effects of the factors (A, B, and C) on the coagulation rate, tensile strength, Young’s modulus, and swelling degree, where it is possible to verify how the average of each response variable is affected by the change in the levels of the independent factors.

All response variables are influenced by the factors under study since no horizontal lines were observed [69]. The coagulation rate (Figure 10a) is negatively affected by the increase in the levels of A and C, that is, by the increase in the molar mass of CS and by the change from ethanol to methanol in the coagulation bath. On the other hand, the acid change (factor B), from acetic to lactic, increases the coagulation rate. The same behavior is observed for tensile strength, with more significant effects than for changes in the type of acid.

Increasing the molar mass of CS (factor A) also negatively affects Young’s modulus (Figure 10c), as does replacing acetic acid with lactic acid (factor B). On the other hand, changing ethanol to methanol (factor C) has a positive effect on this mechanical property of the filaments. Increasing the molar mass and using lactic acid increase the swelling degree of the filaments. Using methanol in the coagulation bath reduces the swelling capacity.

#### 3.4.5. Interaction Between Factors

**Figure 11:**
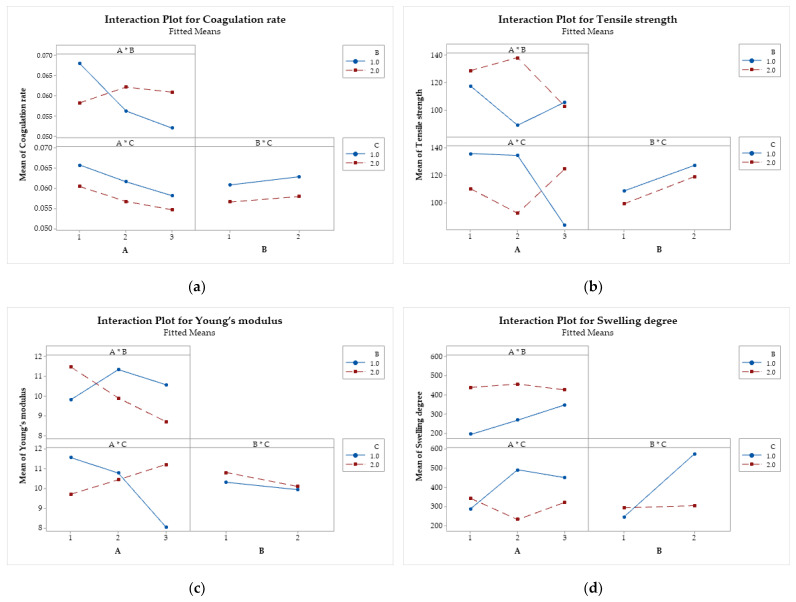
Interaction effects of factors on the (**a**) coagulation rate; (**b**) tensile strength; (**c**) Young’s modulus; and (**d**) swelling degree. Chitosan molar mass (A), acid (B), and coagulation bath (C).

Interaction plots (Figure 11) show the relationship between two independent factors concerning each response variable. One factor is represented on the x-axis, while the other is represented by lines connecting the levels of the second factor. The y-axis indicates the mean of the response variable [68,69]. Parallel lines indicate that the factors do not interact significantly. On the other hand, when the lines are not parallel or intersect, it is an indication that the factors interact with each other.

For the coagulation rate (Figure 11a), only the interaction between factors A and B is significant. This indicates that the relationship between the coagulation rate and the molar mass of chitosan depends on the type of acid used. Figure 11b reveals that the interactions A × B and A × C are significant. In other words, increasing the molar mass of CS increases the tensile strength when methanol is used in the coagulation bath. On the other hand, when ethanol is used instead, the molar mass of CS decreases the resistance of the filaments. The interactions A × B and A × C (Figure 11c) were also significant for Young’s modulus. The modulus decreases with increasing molar mass of CS when lactic acid and ethanol are used, while it tends to increase when acetic acid and methanol are used. For the degree of swelling (Figure 11d), the interactions A × C and B × C were significant, with the effects of molar mass and type of acid depend on the composition of the coagulation bath.

#### 3.4.6. Optimization of the Responses

**Figure 12:**
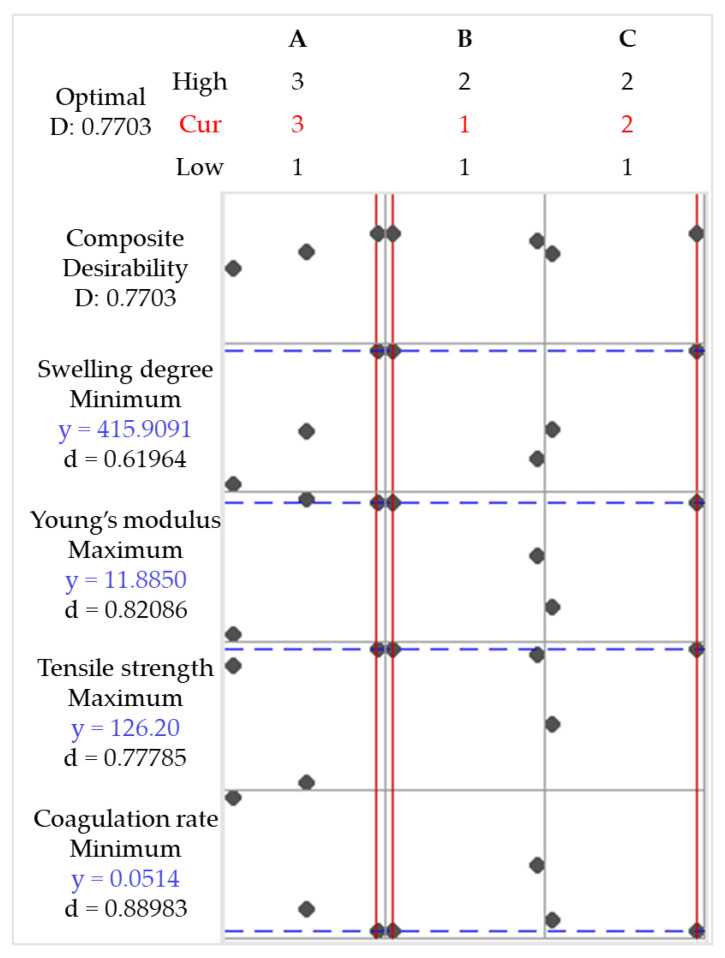
Optimization plot for coagulation rate, tensile strength, Young’s modulus, and swelling degree. Chitosan molar mass (A), acid (B), and coagulation bath (C).

The factorial experimental design was applied to statistically understand the influence of independent factors on the response variables. The data were used to construct mathematical models (Supplementary Material, Table S3) with linear terms and first- and second-order interactions. These models were used to optimize the response variables according to Table S4. The aim was to minimize the coagulation rate and the degree of swelling, in addition to maximizing Young’s modulus and tensile strength. Figure 12 shows the results of applying the optimization conditions.

The conditions A = 3, B = 1, and C = 2 (i.e., high-molecular-weight CS, acetic acid, and methanol in the coagulation bath; sample code 3AAM) represent the selected factor levels that simultaneously optimize the desired responses, with a Desirability value of D = 0.7703. In other words, sample 3AAM is expected to achieve 77.03% of the optimization objective (Table S3). Table 8 presents the fitted responses (Fit), their standard errors (SE Fit), confidence intervals (CI), and prediction intervals (PI) for each response variable. The optimization is successful because it provides reliable predictions for multiple responses. However, variables with larger standard errors and wider intervals (e.g., tensile strength) may require additional model adjustments or greater experimental control to reduce variability.

These findings provide optimized parameters for the fabrication of filaments with controlled properties, which is essential for biomedical applications such as wound dressing manufacturing, tissue engineering, and controlled drug delivery. The ability to simultaneously tune coagulation kinetics, mechanical properties, and water absorption opens up new possibilities for the customization of CS filaments for various advanced applications.

### 3.5. Optimized Sample Characterization

As obtained in the response optimization step, sample 3AAM meets 77.03% of the selected optimization objectives. The sample was characterized by XRD, FT-IR, and SEM, and compared with the other samples obtained from the same CS (A = 3, 200 KDa), and the results are presented below.

#### 3.5.1. X-Ray Diffraction of CS Filaments

**Figure 13:**
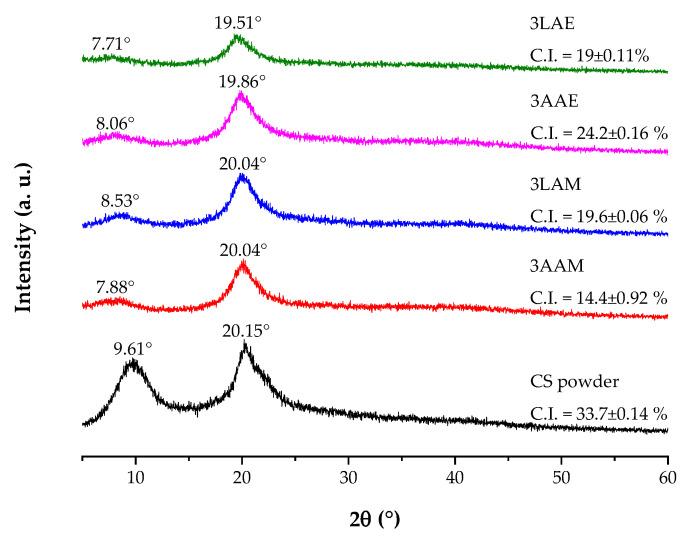
X-ray diffraction patterns of chitosan powder samples and filaments obtained with high molar mass chitosan.

The X-ray diffraction patterns for the CS powder samples and filaments obtained with high molar mass CS are presented in Figure 13. Initially, for the CS powder sample, two diffraction peaks, characteristic of semicrystalline polymers, are observed at 2θ = 10° and 20°. The peak at 10° is commonly attributed to molecular organization, while the peak at 20° reflects the superposition of crystalline and amorphous regions, which is typical of CS [18,70].

In the diffraction patterns of the filaments, there is a reduction in the intensity of the peaks, indicating a decrease in crystallinity after processing. The peak at 2θ ≈ 10° showed slight variations between the samples, with the greatest reduction observed in sample 3LAE. This reduction may be related to the lower capacity of lactic acid to induce molecular organization during filament formation. Studies indicate that acids with more complex chains may hinder the structural reorganization necessary for crystallization [71,72].

The use of acetic acid (samples 3AAM and 3AAE), on the other hand, was found to promote greater molecular reorganization of chitosan. This capacity is related to the efficiency of acetic acid in protonating the amino groups of CS, facilitating the dissolution of the polymer and promoting more homogeneous solutions, which in turn allows for better reorganization during coagulation [73,74,75].

In addition to the influence of the acid used, the type of alcohol in the coagulant bath also influences the structure of the material. Methanol, due to its shorter carbon chain and reduced polarity, induces faster coagulation, limiting the molecular rearrangement necessary for the formation of well-defined crystalline regions [76]. In contrast, ethanol, with greater polarity, favors stronger intermolecular interactions and prolongs the coagulation process, allowing more efficient reorganization and the formation of a more ordered molecular structure [77,78].

The crystallinity index (C.I.), calculated based on the diffraction profiles, is a quantitative indicator of the structural changes in the materials. The CS powder exhibited the highest index (33.7%), evidencing its initial semicrystalline structure. After processing, there was a reduction in the crystallinity indexes of the filaments, reflecting the effect of the dissolution promoted by the acid and the molecular rearrangement during coagulation. Among the processed samples, 3AAE maintained the highest index (24.2%), suggesting that the combination of acetic acid and ethanol favored the formation of crystalline regions. In contrast, the 3LAE sample achieved a lower crystallinity index (19%), even using ethanol, reinforcing that lactic acid induces greater structural disorder, hindering the formation of crystalline regions.

#### 3.5.2. FT-IR of CS Filaments

**Figure 14:**
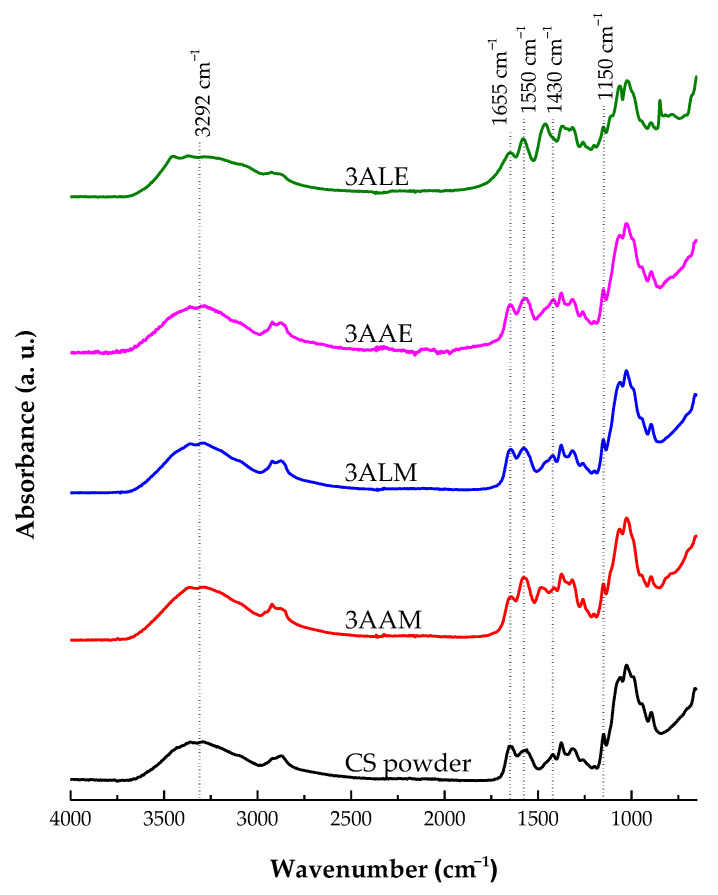
FT-IR spectra of chitosan powder samples and filaments obtained with high molar mass chitosan.

Figure 14 presents the FT-IR spectra of the CS filament and powder samples. Initially, a broad asymmetric band is observed in all samples in the spectral region between 3400 and 2500 cm⁻^1^, which encompasses the C-H stretching modes around 3292 and 2879 cm⁻^1^. At 3292 cm⁻^1^, a band attributed to the stretching of the hydroxyl (O-H) and amino (N-H) groups was identified. This band highlights the presence of intramolecular and intermolecular hydrogen bonds in chitosan [79,80,81]. The intensity of this peak is slightly higher in the samples coagulated with methanol (3LAM and 3AAM) than those coagulated with ethanol (3LAE and 3AAE), which can be attributed to the rearrangement of the hydrophilic structure promoted by methanol as a coagulant.

Furthermore, the peak located at 1655 cm⁻^1^, which corresponds to the stretching vibration of the carbonyl group (C=O) of amide I, showed similar intensities in all samples. This behavior suggests that the configuration of the amide group was not significantly altered by the different acids (lactic and acetic) or coagulants (methanol and ethanol) used. The band at 1550 cm⁻^1^, associated with the deformation vibrations of the N-H groups of amide II of CS, reinforces the preservation of the amide structure [80,82,83].

At 1430 cm⁻^1^, deformation of the C-H bonds in glycosidic rings was observed, whose intensity was higher in the samples coagulated with ethanol (3LAE and 3AAE), which may indicate stronger interactions between ethanol and the glycosidic groups of chitosan. On the other hand, methanol appears to better preserve the structural integrity of the polymer. The band at 1150 cm⁻^1^, attributed to the asymmetric stretching of C-O-C of the glycosidic bridges, did not show significant differences in intensity between the samples treated with lactic or acetic acid, nor between those coagulated with methanol or ethanol [84,85,86]. These results suggest the preservation of the polymeric structure of CS under all conditions evaluated.

#### 3.5.3. Scanning Electron Microscopy of CS Filaments

**Figure 15:**
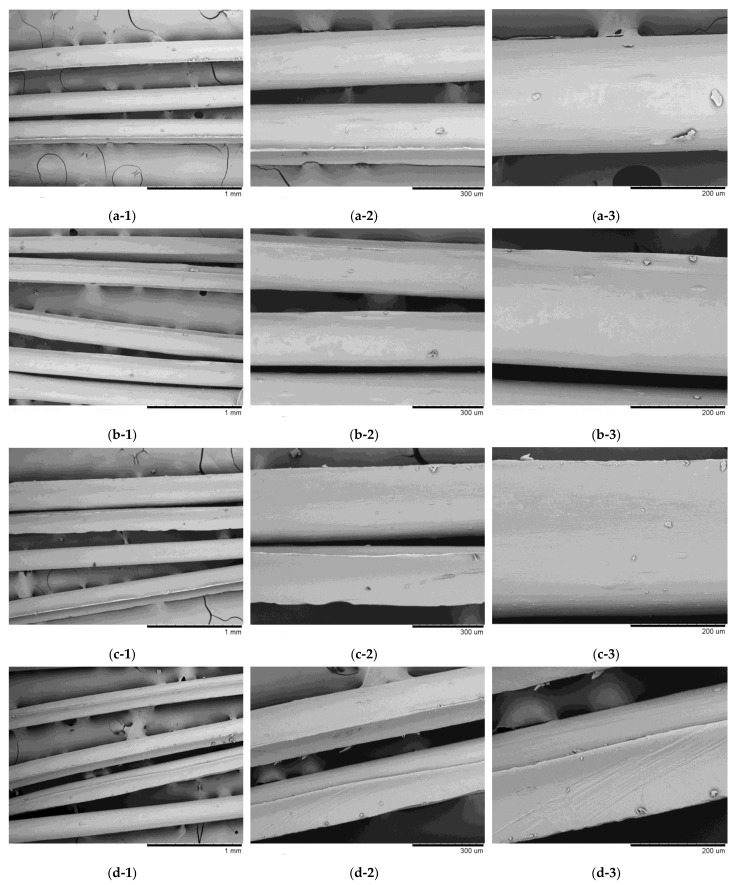
Scanning electron microscopy (SEM) images of filament samples obtained with high molar mass chitosan: (**a**) 3LAE (1: 100, 2: 250, and 3: 500×); (**b**) 3AAE (1: 100, 2: 250, and 3: 500×); (**c**) 3LAM (1: 100, 2: 250, and 3: 500×); (**d**) 3AAM (1: 100, 2: 250, and 3: 500×).

Figure 15 shows the SEM images of the filament samples obtained with high molar mass CS. The samples were similar to each other, with a relatively homogeneous and well-oriented surface free of pores and surface depressions. The filaments also exhibited longitudinal grooves, probably due to the spinning profile or the support used for drying.

The filaments obtained with ethanol in the coagulation bath (Figure 15a,b) exhibited smoother and more homogeneous morphologies, compared to those obtained with methanol. This effect can be attributed to the fact that ethanol increases the coagulation rate of chitosan solutions, as shown in the main effects graph (Figure 10a). Furthermore, among the samples obtained with high molar mass chitosan, sample 3LAE presented a higher coagulation rate (Table 3), which may explain the greater homogeneity observed. In this case, both the use of lactic acid and ethanol tend to increase the coagulation rate (Figure 10a), resulting in a positive synergistic effect for this sample. Faster coagulation rates tend to result in a smooth and more homogeneous surface structure, due to the high speed of filament formation [87,88].

The samples obtained with methanol (Figure 15c, d) were rougher and had more pronounced grooves, with sample 3AAM being the most irregular. This result is also aligned with that observed in Figure 10a, where acetic acid and methanol tend to reduce the coagulation rate of the systems. According to Dobrovolskaya et al. [89], to obtain an oriented system of macromolecules during wet spinning, it is essential to maximize intermolecular contacts, providing laminarity in the flow during extrusion and in the precipitation bath. In addition, the mobility of macromolecular chains is essential to facilitate the development of more compact structures, which ensures the orientation and regularity of macromolecules. These conditions seem to be achieved more efficiently when using lactic acid, resulting in filaments with better surface quality. This behavior can be attributed to the interaction of lactic acid with the polymeric chains of chitosan, promoting a more uniform structural rearrangement and reducing viscosity at the time of spinning [53,56,57,90]. On the other hand, the molecular interactions of acetic acid with CS can promote its gelation and consequent increase in viscosity, which negatively influences the spinning process and the morphology of the resulting filaments [55].

These findings are in accordance with the work of Gnus [91], who demonstrated that the type of solvent significantly influences the properties of CS membranes.

## 4. Conclusions

A full factorial design was successfully employed to demonstrate how the coagulation process, mechanical properties, and swelling behavior of chitosan (CS) filaments are influenced by the CS molar mass, the acid used as a solvent, and the composition of the coagulation bath.

It was demonstrated that increasing the chitosan molar mass tends to decrease the coagulation rate of CS solutions, as does the use of methanol in the coagulation bath instead of ethanol. Furthermore, solubilization of CS in lactic acid tends to increase the coagulation rate, compared to acetic acid.

Mechanical tests revealed that CS with higher molar mass produces filaments with larger diameters but does not necessarily improve tensile strength due to reduced homogeneity. Filaments prepared with lactic acid exhibited superior mechanical properties to those prepared with acetic acid. The swelling behavior was mainly influenced by the acid solvent and coagulation bath composition.

Factorial design analysis highlighted the significant contributions of the molar mass, acid solvent, and coagulation bath composition to all response variables. The models for coagulation rate and swelling degree showed high predictive power, while the tensile strength and Young’s modulus models indicated areas for refinement. Interaction effects, such as CS molar mass with acid type or coagulation bath composition, were critical determinants of the observed behaviors.

The results obtained are of great practical importance for improving the wet spinning technique of CS filaments and for their applications in various areas, especially biomedicine:

- Adjusting the CS molar mass to control fiber diameter without compromising mechanical strength;
- Choosing the appropriate acid solvent (e.g., acetic acid for greater crystallinity or lac-tic acid for greater flexibility and water absorption) depending on the desired application;
- Changing the composition of the coagulation bath to obtain more homogeneous or rough surfaces, directly influencing its application in tissue engineering;
- Improving the mechanical strength and crystallinity of the filaments, making them more durable for sutures and cellular scaffolds;
- Adjusting the absorption and swelling rate, allowing greater control over drug release or biodegradation in contact with biological tissues;
- Adjustments in the manufacture of CS filaments without the need for crosslinking or reinforcement with other materials.

## Figures and Tables

**Table 1:** Factors and their levels evaluated in the experimental design.

Levels	A—Chitosan Molar Mass	B—Acid	C—Coagulation Bath
1	83 KDa	Acetic	NaOH 0.5M + Ethanol
2	155 KDa	Lactic	NaOH 0.5M + Methanol
3	200 KDa	-	-

**Table 2:** Treatments performed and run order applied.

Standard Order	Run Order	Sample Code *	A	B	C
21	1	3AAE	3	1	1
14	2	2AAM	2	1	2
20	3	1LAM	1	2	2
22	4	3AAM	3	1	2
13	5	2AAE	2	1	1
17	6	1AAE	1	1	1
18	7	1AAM	1	1	2
8	8	1LAM	1	2	2
24	9	3LAM	3	2	2
9	10	3AAE	3	1	1
23	11	3LAE	3	2	1
19	12	1LAE	1	2	1
15	13	2LAE	2	2	1
7	14	1LAE	1	2	1
11	15	3LAE	3	2	1
16	16	2LAM	2	2	2
5	17	1AAE	1	1	1
12	18	3LAM	3	2	2
2	19	2AAM	2	1	2
10	20	3AAM	3	1	2
4	21	2LAM	2	2	2
3	22	2LAE	2	2	1
6	23	1AAM	1	1	2
1	24	2AAE	2	1	1

* Coding: the numbers 1, 2, and 3 represent the molar mass of chitosan (low, medium, and high, respectively); the acronyms AA and LA are the solvents used, acetic acid and lactic acid, respectively; the letter E refers to ethanol, while M refers to methanol in the coagulant bath.

**Table 3:** Average coagulation rates of the evaluated systems.

Sample Code	Coagulation Rate (mm/s^1/2^)
1AAE	0.0710 ± 0.00198
2AAE	0.0589 ± 0.00438
3AAE	0.0525 ± 0.00431
1AAM	0.0648 ± 0.00205
2AAM	0.0535 ± 0.00092
3AAM	0.0514 ± 0.00368
1LAE	0.0604 ± 0.00262
2LAE	0.0643 ± 0.00283
3LAE	0.0637 ± 0.00106
1LAM	0.0560 ± 0.00010
2LAM	0.0599 ± 0.00375
3LAM	0.0579 ± 0.00163

**Table 4:** Average values of mechanical properties and diameter obtained for the evaluated systems.

SAMPLE CODE	Diameter (µm)	Tensile Strength (MPa)	Young’s Modulus (GPa)	Strain at Fracture (%)
1AAE	203.0 ± 33.0	118 ± 34.4	11.0 ± 0.37	3.5 ± 0.71
2AAE	213.3 ± 16.03	123 ± 21.1	10.7 ± 2.88	4.3 ± 0.42
3AAE	242.7 ± 9.43	85 ± 12.8	9.3 ± 0.56	3.4 ± 0.28
1AAM	207.5 ± 8.25	117 ± 12.8	8.6 ± 0.93	3.9 ± 0.25
2AAM	235.3 ± 2.83	55 ± 61.0	12.0 ± 0.05	4.3 ± 0.60
3AAM	225.2 ± 20.51	126 ± 4.3	11.9 ± 0.43	3.8 ± 0.22
1LAE	185.2 ± 6.36	154 ± 7.1	12.1 ± 1.22	5.7 ± 0.73
2LAE	206.3 ± 33.47	146 ± 18.8	10.9 ± 0.99	4.3 ± 0.11
3LAE	245.7 ± 7.54	82 ± 1.3	6.8 ± 0.18	4.1 ± 0.30
1LAM	198.5 ± 9.19	103 ± 4.1	10.8 ± 0.61	3.9 ± 0.59
2LAM	214.3 ± 16.03	131 ± 0.8	8.9 ± 0.44	4.3 ± 0.07
3LAM	223.3 ± 12.73	123 ± 6.9	10.6 ± 0.11	4.3 ± 0.05

**Table 5:** ANOVA results for coagulation rate and tensile strength.

**Coagulation Rate**						
Source *	**DF**	**Contribution**	**Adj SS**	**Adj MS**	**F-Value**	***p*** **-Value**
Model	11	88.77%	0.000735	0.000067	8.62	0.000
A	2	21.66%	0.000179	0.000090	11.57	0.002
B	1	2.07%	0.000017	0.000017	2.22	0.162
C	1	14.95%	0.000124	0.000124	15.97	0.002
A × B	2	48.10%	0.000398	0.000199	25.69	0.000
A × C	2	0.44%	0.000004	0.000002	0.23	0.795
B × C	1	0.08%	0.000001	0.000001	0.08	0.780
A × B × C	2	1.46%	0.000012	0.000006	0.78	0.479
Error	12	11.23%	0.000093	0.000008		
**Tensile Strength**						
Source *	**DF**	**Contribution**	**Adj SS**	**Adj MS**	**F-Value**	***p*** **-Value**
Model	11	73.74%	17,339.9	1576.35	3.06	0.033
A	2	6.08%	1430.7	715.33	1.39	0.286
B	1	9.33%	2195.0	2194.98	4.27	0.061
C	1	1.94%	455.9	455.88	0.89	0.365
A × B	2	12.42%	2919.4	1459.72	2.84	0.098
A × C	2	32.57%	7657.9	3828.94	7.44	0.008
B × C	1	0.01%	2.0	2.01	0.00	0.951
A × B × C	2	11.39%	2679.0	1339.52	2.60	0.115
Error	12	26.26%	6173.6	514.47		

* Chitosan molar mass (A), acid (B), and coagulation bath (C). Linear factors (A, B, and C) and second- (A × B, A × C, and B × C) and third-order interactions (A × B × C).

**Table 6:** ANOVA results for Young’s modulus and swelling degree.

Young’s Modulus						
Source *	DF	Contribution	Adj SS	Adj MS	F-Value	*p*-Value
Model	11	81.11%	553.642	50.331	4.68	0.007
A	2	7.70%	52.561	26.281	2.45	0.129
B	1	2.57%	17.550	17.550	1.63	0.225
C	1	0.93%	0.6370	0.6370	0.59	0.456
A × B	2	21.80%	148.814	74.407	6.93	0.010
A × C	2	38.85%	265.204	132.602	12.34	0.001
B × C	1	0.24%	0.1650	0.1650	0.15	0.702
A × B × C	2	9.01%	61.492	30.746	2.86	0.096
Error	12	18.89%	128.921	10.743		
**Swelling Degree**						
Source *	**DF**	**Contribution**	**Adj SS**	**Adj MS**	**F-Value**	***p*** **-Value**
Model	11	94.76%	701,734	63,794	19.72	0.000
A	2	2.78%	20,583	10,292	3.18	0.078
B	1	23.45%	173,635	173,635	53.68	0.000
C	1	9.93%	73,524	73,524	22.73	0.000
A × B	2	3.87%	28,660	14,330	4.43	0.036
A × C	2	13.49%	99,891	49,946	15.44	0.000
B × C	1	20.37%	150,871	150,871	46.64	0.000
A × B × C	2	20.87%	154,569	77,284	23.89	0.000
Error	12	5.24%	38,818	3235		

* Chitosan molar mass (A), acid (B), and coagulation bath (C). Linear factors (A, B, and C) and second- (A × B, A × C, and B × C) and third-order interactions (A × B × C).

**Table 7:** Adjustment parameters of regression models for response variables.

Response	S	R-sq	R-sq(adj)	PRESS	AICc	BIC
Coagulation Rate	0.0027835	88.77%	78.47%	0.0003719	−168.56	−189.65
Tensile Strength	226.819	73.74%	49.68%	24694.4	263.71	242.62
Young’s modulus	103.650	81.11%	63.80%	515.682	115.59	94.51
Swelling degree	568.756	94.76%	89.95%	155272	307.84	286.75

**Table 8:** Multiple prediction for response variables.

Response	Fit	SE Fit	95% CI	95% PI
Swelling degree (%)	415.9	40.2	(328.3, 503.5)	(264.1, 567.7)
Young’s modulus (GPa)	11.885	0.733	(10.288, 13.482)	(9.119, 14.651)
Tensile strength (MPa)	126.2	16.0	(91.3, 161.1)	(65.7, 186.7)
Coagulation rate (mm/s^1/2^)	0.05140	0.00197	(0.04711, 0.05569)	(0.04397, 0.05883)

## Data Availability

The datasets presented in this article are not readily available because the data are part of an ongoing study. Requests to access the datasets should be directed to the corresponding authors.

## Supplementary Materials

The following supporting information can be downloaded at: https://www.mdpi.com/article/10.3390/polym17070927/s1, Figure S1. Temporal evolution of the coagulated thicknesses (coagulation profile) of the evaluated systems; Table S1. Raw data for full factorial design; Table S2. Results of linear regressions for the coagulation rate; Table S3. Prediction models for response variables; Table S4. Adjustment parameters for response optimization.
