# Supplementary material for: How Molar Mass, Acid Type, and Coagulation Bath Composition Influence Coagulation Kinetics, Mechanical Properties, and Swelling Behavior of Chitosan Filaments: A Full Factorial Approach
Source: Polymers (Basel). 2025 Mar 29;17(7):927. doi: 10.3390/polym17070927 (PMC11991260; doi:10.3390/polym17070927)
Supplement: Supplementary file 1 [file polymers-17-00927-s001.zip › polymers-3504698-supplementary.pdf]

Supplementary material

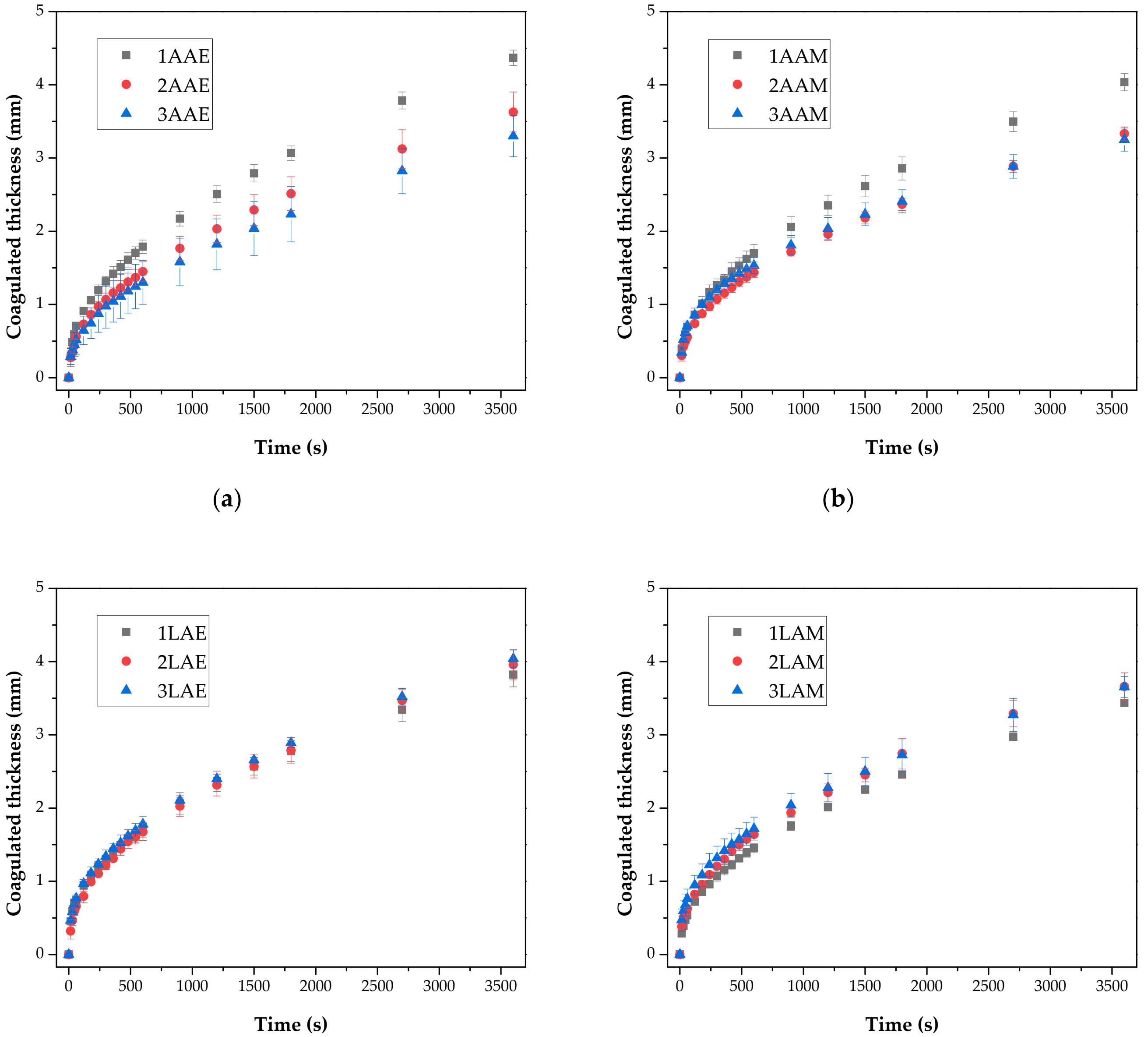

Figure S1. Temporal evolution of the coagulated thicknesses (coagulation profile) of the evaluated systems. Samples prepared with chitosan of variable molar mass (3, 2 and 1) and: (**a**) Acetic acid and ethanol; (**b**) Acetic acid and methanol; (**c**) Lactic acid and ethanol; (**d**) Lactic acid and methanol.

Table S1. Raw data for full factorial design.

Standard order	Run order	Point type	Blocks	A	B	C	Coagulation rate	Tensile strength	Young's modulus	Swelling degree
21	1	1	1	3	1	1	0.0556	94.33	9.67	303.3
14	2	1	1	2	1	2	0.0529	11.56	11.94	286.2
20	3	1	1	1	2	2	0.0560	100.65	10.40	581.8
22	4	1	1	3	1	2	0.0540	123.19	12.19	450.0
13	5	1	1	2	1	1	0.0558	138.11	12.75	240.0
17	6	1	1	1	1	1	0.0696	93.41	10.75	203.4
18	7	1	1	1	1	2	0.0634	126.51	9.28	196.8
8	8	1	1	1	2	2	0.0560	106.45	11.27	421.4
24	9	1	1	3	2	2	0.0568	118.33	10.50	222.2
9	10	1	1	3	1	1	0.0495	76.26	8.88	261.8
23	11	1	1	3	2	1	0.0630	81.36	6.98	558.3
19	12	1	1	1	2	1	0.0586	148.88	13.01	375.0
15	13	1	1	2	2	1	0.0663	158.94	10.18	809.7
7	14	1	1	1	2	1	0.0623	158.94	11.28	381.3
11	15	1	1	3	2	1	0.0645	83.23	6.73	686.1
16	16	1	1	2	2	2	0.0626	131.28	9.26	200.0
5	17	1	1	1	1	1	0.0724	142.12	11.28	200.0
12	18	1	1	3	2	2	0.0591	128.11	10.66	242.3
2	19	1	1	2	1	2	0.0542	97.87	12.01	276.7
10	20	1	1	3	1	2	0.0488	129.21	11.58	381.8
4	21	1	1	2	2	2	0.0573	130.10	8.63	174.2
3	22	1	1	2	2	1	0.0623	132.40	11.58	647.2
6	23	1	1	1	1	2	0.0663	108.35	7.96	181.2
1	24	1	1	2	1	1	0.0620	108.23	8.68	276.9

Table S2. Results of linear regressions for the coagulation rate.

Run order	Sample code	Linear regression	Coagulation Rate	R^2
1	3AAE	y = 0.0556x + 0.161	0.0556	0.9974
2	2AAM	y = 0.0529x + 0.0917	0.0529	0.9987
3	1ALM	y = 0.056x + 0.055	0.0560	0.9995
4	3AAM	y = 0.054x + 0.2456	0.0540	0.9924
5	2AAE	y = 0.0558x + 0.0204	0.0558	0.9977
6	1AAE	y = 0.0696x + 0.0576	0.0696	0.9969
7	1AAM	y = 0.0634x + 0.0871	0.0634	0.9985
8	1ALM	y = 0.056x + 0.1104	0.0560	0.9985
9	3ALM	y = 0.0568x + 0.1897	0.0568	0.9966
10	3AAE	y = 0.0495x - 0.0582	0.0495	0.9914
11	3ALE	y = 0.063x + 0.2007	0.0630	0.9972
12	1ALE	y = 0.0586x + 0.2039	0.0586	0.9965
13	2ALE	y = 0.0663x + 0.1249	0.0663	0.9987
14	1ALE	y = 0.0623x + 0.271	0.0623	0.9947
15	3ALE	y = 0.0645x + 0.2279	0.0645	0.9961
16	2ALM	y = 0.0626x + 0.1264	0.0626	0.9978
17	1AAE	y = 0.0724x + 0.0908	0.0724	0.9994
18	3ALM	y = 0.0591x + 0.3553	0.0591	0.9905
19	2AAM	y = 0.0542x + 0.1495	0.0542	0.9981
20	3AAM	y = 0.0488x + 0.2544	0.0488	0.9898
21	2ALM	y = 0.0573x + 0.1749	0.0573	0.9966
22	2ALE	y = 0.0623x + 0.0794	0.0623	0.9982
23	1AAM	y = 0.0663x + 0.1596	0.0663	0.9984
24	2AAE	y = 0.062x + 0.0535	0.0620	0.9989

Table S3. Prediction models for response variables.

**Coagulation rate**
0.059554 + 0.003521 A_1 - 0.000379 A_2 - 0.003142 A_3 - 0.000846 B_1 + 0.000846 B_2 + 0.002271 C_1 - 0.002271 C_2 + 0.005696 A*B_1 1 - 0.005696 A*B_1 2 - 0.002104 A*B_2 1 + 0.002104 A*B_2 2 - 0.003592 A*B_3 1 + 0.003592 A*B_3 2 + 0.000379 A*C_1 1 - 0.000379 A*C_1 2 + 0.000154 A*C_2 1 - 0.000154 A*C_2 2 - 0.000533 A*C_3 1 + 0.000533 A*C_3 2 - 0.000162 B*C_1 1 + 0.000162 B*C_1 2 + 0.000162 B*C_2 1 - 0.000162 B*C_2 2 + 0.000588 A*B*C_1 1 1 - 0.000588 A*B*C_1 1 2 - 0.000588 A*B*C_1 2 1 + 0.000588 A*B*C_1 2 2 + 0.000412 A*B*C_2 1 1 - 0.000412 A*B*C_2 1 2 - 0.000412 A*B*C_2 2 1 + 0.000412 A*B*C_2 2 2 - 0.001000 A*B*C_3 1 1 + 0.001000 A*B*C_3 1 2 + 0.001000 A*B*C_3 2 1 - 0.001000 A*B*C_3 2 2
**Tensile Strength**
113.66 + 9.50 A_1 - 0.10 A_2 - 9.41 A_3 - 9.56 B_1 + 9.56 B_2 + 4.36 C_1 - 4.36 C_2 + 4.00 A*B_1 1 - 4.00 A*B_1 2 - 15.06 A*B_2 1 + 15.06 A*B_2 2 + 11.06 A*B_3 1 - 11.06 A*B_3 2 + 8.32 A*C_1 1 - 8.32 A*C_1 2 + 16.50 A*C_2 1 - 16.50 A*C_2 2 - 24.82 A*C_3 1 + 24.82 A*C_3 2 + 0.29 B*C_1 1 - 0.29 B*C_1 2 - 0.29 B*C_2 1 + 0.29 B*C_2 2 - 12.80 A*B*C_1 1 1 + 12.80 A*B*C_1 1 2 + 12.80 A*B*C_1 2 1 - 12.80 A*B*C_1 2 2 + 13.08 A*B*C_2 1 1 - 13.08 A*B*C_2 1 2 - 13.08 A*B*C_2 2 1 + 13.08 A*B*C_2 2 2 - 0.28 A*B*C_3 1 1 + 0.28 A*B*C_3 1 2 + 0.28 A*B*C_3 2 1 - 0.28 A*B*C_3 2 2
**Young's modulus**
10.310 + 0.343 A_1 + 0.318 A_2 - 0.662 A_3 + 0.270 B_1 - 0.270 B_2 - 0.163 C_1 + 0.163 C_2 - 1.107 A*B_1 1 + 1.107 A*B_1 2 + 0.446 A*B_2 1 - 0.446 A*B_2 2 + 0.661 A*B_3 1 - 0.661 A*B_3 2 + 1.089 A*C_1 1 - 1.089 A*C_1 2 + 0.332 A*C_2 1 - 0.332 A*C_2 2 - 1.421 A*C_3 1 + 1.421 A*C_3 2 - 0.083 B*C_1 1 + 0.083 B*C_1 2 + 0.083 B*C_2 1 - 0.083 B*C_2 2 + 0.354 A*B*C_1 1 1 - 0.354 A*B*C_1 1 2 - 0.354 A*B*C_1 2 1 + 0.354 A*B*C_1 2 2 - 0.716 A*B*C_2 1 1 + 0.716 A*B*C_2 1 2 + 0.716 A*B*C_2 2 1 - 0.716 A*B*C_2 2 2 + 0.362 A*B*C_3 1 1 - 0.362 A*B*C_3 1 2 - 0.362 A*B*C_3 2 1 + 0.362 A*B*C_3 2 2
**Swelling degree**
356.6 - 39.0 A_1 + 7.3 A_2 + 31.7 A_3 - 85.1 B_1 + 85.1 B_2 + 55.3 C_1 - 55.3 C_2 - 37.2 A*B_1 1 + 37.2 A*B_1 2 - 8.9 A*B_2 1 + 8.9 A*B_2 2 + 46.1 A*B_3 1 - 46.1 A*B_3 2 - 83.0 A*C_1 1 + 83.0 A*C_1 2 + 74.2 A*C_2 1 - 74.2 A*C_2 2 + 8.8 A*C_3 1 - 8.8 A*C_3 2 - 79.3 B*C_1 1 + 79.3 B*C_1 2 + 79.3 B*C_2 1 - 79.3 B*C_2 2 + 113.3 A*B*C_1 1 1 - 113.3 A*B*C_1 1 2 - 113.3 A*B*C_1 2 1 + 113.3 A*B*C_1 2 2 - 61.8 A*B*C_2 1 1 + 61.8 A*B*C_2 1 2 + 61.8 A*B*C_2 2 1 - 61.8 A*B*C_2 2 2 - 51.5 A*B*C_3 1 1 + 51.5 A*B*C_3 1 2 + 51.5 A*B*C_3 2 1 - 51.5 A*B*C_3 2 2

Table S4. Adjustment parameters for response optimization.

Response	Goal	Lower	Target	Upper	Weight	Importance
Swelling degree	Minimum	-	174.194	809.677	1	1
Young's modulus	Maximum	6.73	13.010	-	1	1
Tensile strength	Maximum	11.56	158.940	-	1	1
Coagulation rate	Minimum	-	0.049	0.072	1	1
